# A Rare Case of Catheter Obstruction in Peritoneal Dialysis Due to Entrapment of Oviductal Fimbriae

**DOI:** 10.7759/cureus.69131

**Published:** 2024-09-10

**Authors:** Karen D Gómez-Arciniega, Daniel A Ángel-Montoya, Héctor A Benítez-Jauregui, José L Mejía-Ramírez, Cesar O Cortes-González

**Affiliations:** 1 General Surgery, Centro Médico Nacional de Occidente, Instituto Mexicano del Seguro Social, Guadalajara, MEX

**Keywords:** catheter obstruction, chronic kidney disease, fallopian tube, peritoneal dialysis, tenckhoff catheter

## Abstract

Peritoneal dialysis is a renal replacement therapy modality used in patients with end-stage chronic kidney disease. Peritoneal dialysis catheters can present complications such as infections, leaks, catheter kinking, and mechanical obstruction. The obstruction can be caused by fibrin, adhesions, or entrapment of intraperitoneal organs; among them, the most frequent is the omentum. Entrapment of the fallopian tube fimbriae is an extremely rare complication, requiring early surgical attention.

We present the case of a 65-year-old woman with chronic kidney disease on home peritoneal dialysis, who had a Tenckhoff catheter placed three months earlier. She presented to the Emergency Department with abdominal pain and decreased outflow from her catheter. An abdominal CT scan showed a catheter located in the right iliac fossa, mechanical obstruction was suspected, and open abdominal surgery was performed. During the surgery, the fimbriae of the right fallopian tube were found to be trapped in the Tenckhoff catheter. The fallopian tube was freed, and the catheter was adequately repositioned. The patient evolved satisfactorily; peritoneal dialysis was reestablished after two weeks, with no recurrence of long-term entrapment.

## Introduction

In patients with end-stage chronic kidney disease, peritoneal dialysis is an alternative to hemodialysis, which is a renal replacement therapy modality that can be performed at home, provides patient autonomy, and helps to improve quality of life [1].

For peritoneal dialysis to be successful as a form of renal replacement therapy, it requires the peritoneal catheter to be functional over the long term [2,3].

Complications that can occur with a peritoneal dialysis catheter include exit site infection, peritonitis, hernias, leaks, tip malposition, and mechanical obstruction of the catheter [4].

Peritoneal dialysis catheters are obstructed in up to 2-36% of patients. The primary cause of dialysis catheter obstruction is omental entrapment; however, other intra-abdominal tubular structures can also cause blockage of the catheter lumen, such as the appendix and fallopian tubes. Reports of obstruction by oviductal fimbriae are very infrequent [5].

Catheter obstruction results in ineffective peritoneal dialysis requiring urgent attention. Early surgical exploration is necessary for patients with peritoneal dialysis catheter dysfunction to avoid potentially serious complications [4,6].

## Case presentation

The patient is a 65-year-old woman with a history of systemic arterial hypertension, insulin-dependent type 2 diabetes mellitus, and stage 5 chronic kidney disease with ambulatory peritoneal dialysis, and a surgical history of placement of a Tenckhoff peritoneal dialysis catheter three months earlier.

She came to the Emergency Department with 72 hours of slow flow during the infusion of the dialysate solution, as well as difficulty in drainage without recovering the infused volume, in addition to presenting with abdominal pain in the right iliac fossa for 24 hours. On admission to the Emergency Department, her vital signs were stable, and she was afebrile. Physical examination revealed a soft abdomen but very painful on palpation of the right iliac fossa and abdominal rigidity.

**Figure 1:**
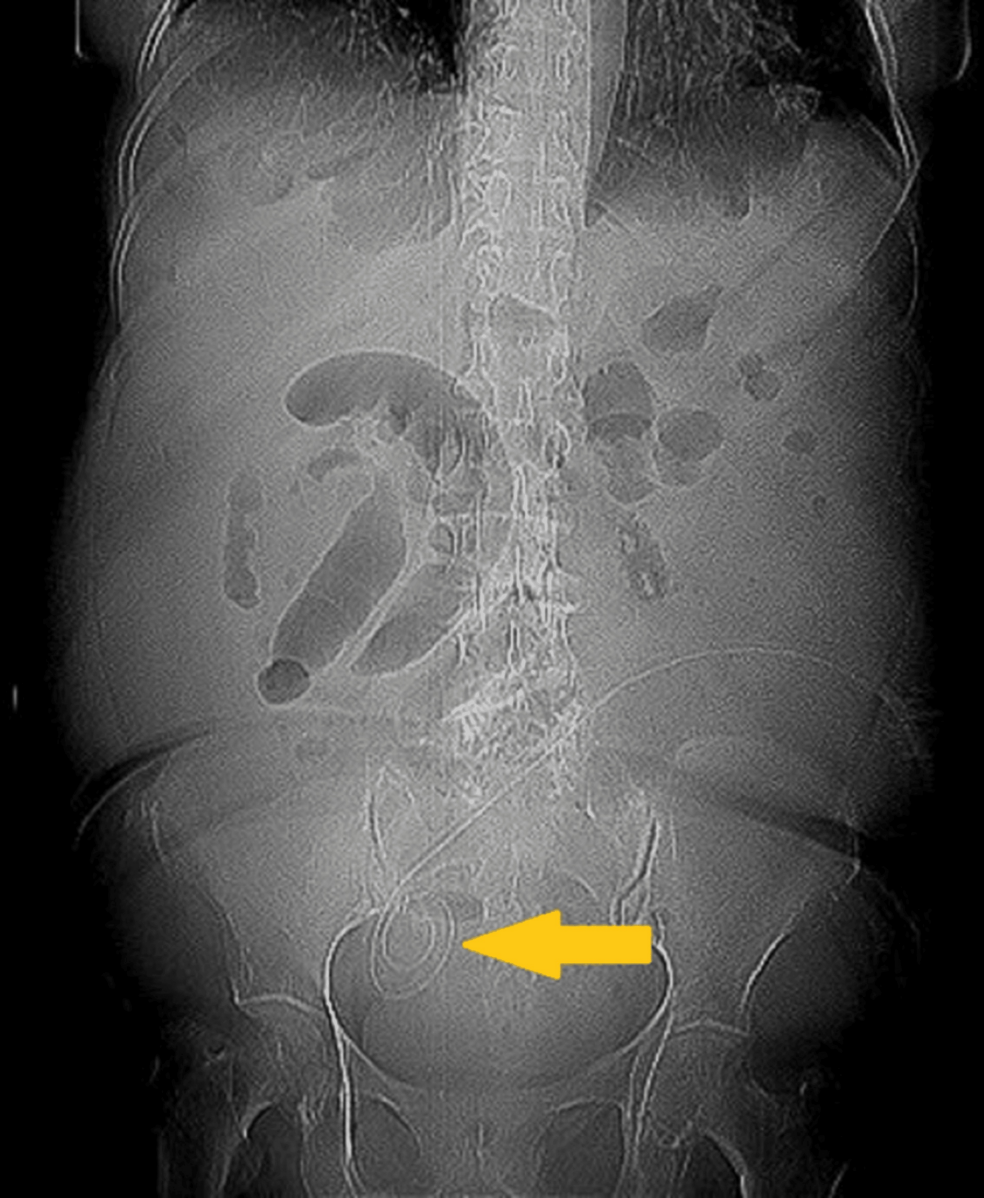
Abdominal X-ray The yellow arrow shows the location of the Tenckhoff catheter in the right iliac fossa, not twisted or bent.

**Figure 2:**
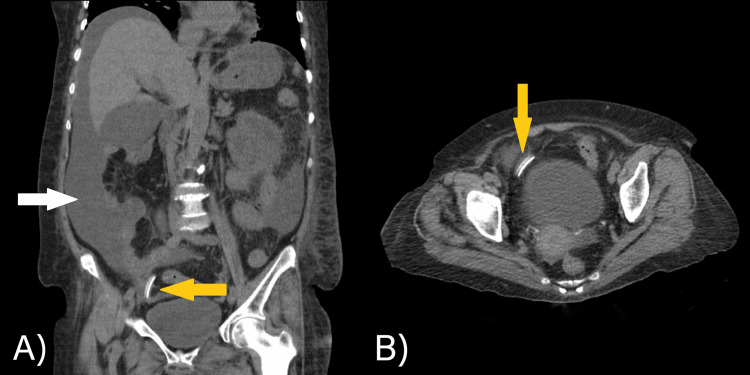
Abdominal CT scan: A) coronal section and B) axial section The yellow arrows show the location of the Tenckhoff catheter in the right iliac fossa. The white arrow shows free intra-abdominal fluid.

A peritoneal fluid culture was performed and was negative. An X-ray showed that the catheter was not kinked (Figure 1). A CT scan of the abdomen and pelvis showed a peritoneal catheter located in the right iliac fossa and free fluid (Figure 2).

**Figure 3:**
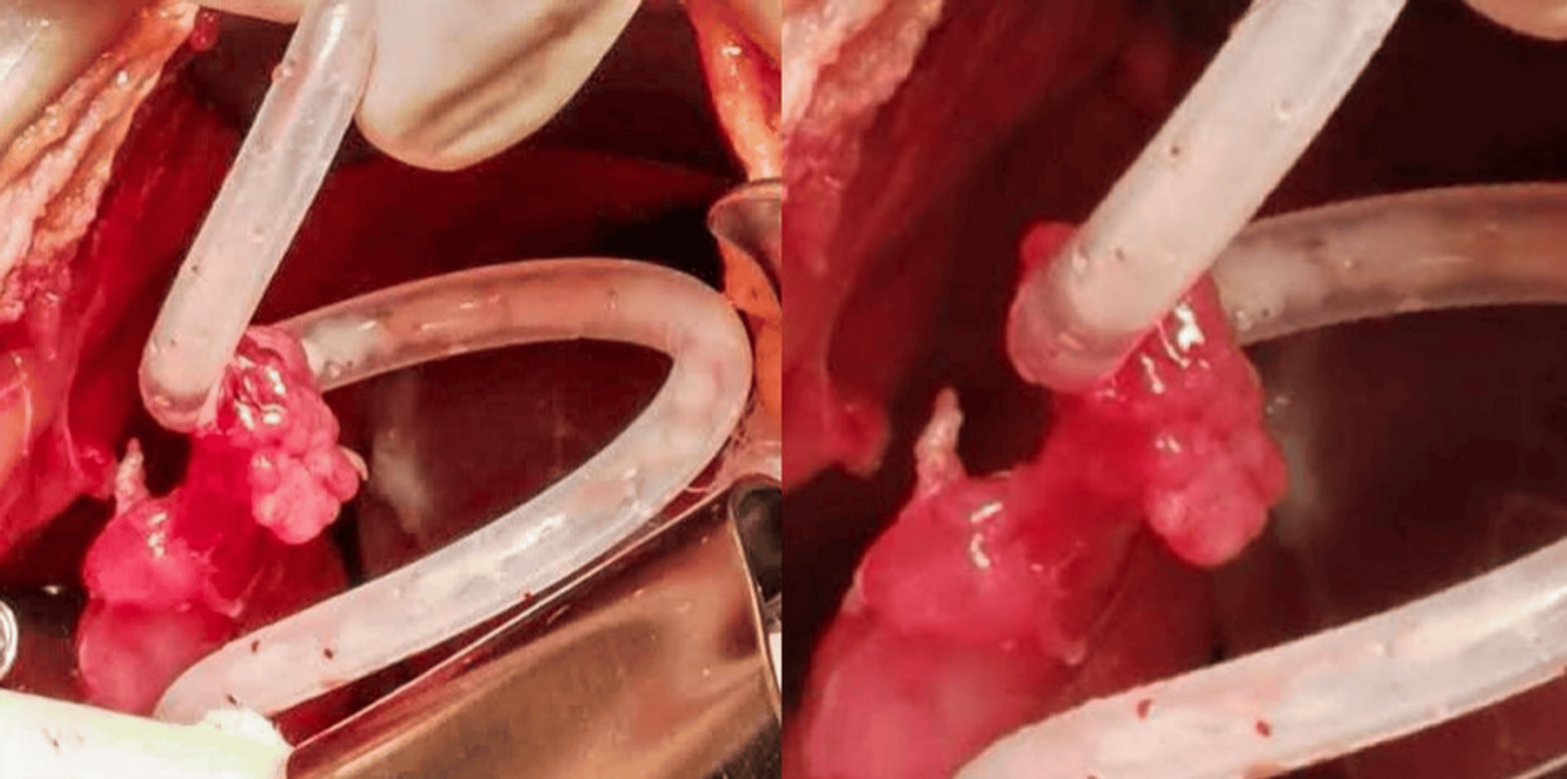
Intraoperative photograph: entrapment of the fallopian tube in the catheter Both photographs show the fimbriae of the right fallopian tube are seen trapped in the fenestrae of the catheter, obstructing its lumen.

Mechanical obstruction of the catheter was suspected, and due to the presence of signs of peritoneal irritation, it was decided to perform an exploratory minilaparotomy. Surgical intervention was performed, during which the erythematous and rotated right fallopian tube was identified over the Tenckhoff catheter; its fimbriae had penetrated the catheter fenestration, blocking the drainage orifices (Figure 3). The fimbriae were then released from the catheter, and the right fallopian tube was carefully placed in its usual location. The catheter was repositioned in the pelvis, and its functionality was corroborated with the instillation of a dialyzing solution.

The patient evolved favorably, without developing post-surgical complications. Peritoneal dialysis was restarted two weeks after surgery, showing adequate infusion and drainage volumes. She was followed by the general surgery outpatient clinic, with no recurrence of Tenckhoff catheter obstruction two years after surgery.

## Discussion

One of the main complications of peritoneal dialysis is a malfunctioning catheter. Causes of peritoneal catheter dysfunction can include kinking, displacement of the catheter, constipation, fibrin deposition, intraperitoneal adhesions caused by infection, and obstruction by intraperitoneal structures [5,6].

Catheter kinking at the subcutaneous level usually manifests as slow dialysis fluid velocity, including inflow and outflow. This presentation is similar to that which occurs with obstruction of the catheter lumen by fibrin or blood clots, while entrapment by adhesions or viscera usually results in outflow failure, causing the volume of drainage fluid to be less than the inflow [3].

Entrapment of the omentum is one of the most frequent causes of peritoneal dialysis catheter obstruction, but there are other intraperitoneal structures that, in rare cases, can also become entrapped and compromise catheter flow, such as the appendix or fallopian tube. Entrapment of visceral structures has potentially serious morbidity [4,5].

Catheter obstruction secondary to entrapment of the fallopian tube fimbriae and their subsequent growth into the lumen is a clearly infrequent complication. Obstructions of this type have been described most frequently on the right side [7,8].

Some of the triggering factors that may explain the entrapment of the oviductal fimbriae and subsequent catheter obstruction are the bioincompatibility of the catheter materials, adhesions associated with previous surgeries, previous episodes of peritonitis, the absence of normal fimbrial movements in uremic patients, and, in the case of girls, the small size of the organs in relation to the catheter [6,9-11].

The peritoneal cavity and the uterine cavity are connected by the fallopian tubes, but free fluid from the peritoneal cavity does not access the uterine cavity because of high resistance to flow. However, if the tip of the catheter is close to the salpingium, this resistance to flow can be overcome, which explains the vaginal leakage of dialysis fluid that has been reported in some patients with fallopian tube entrapment by the peritoneal catheter [6].

In addition to vaginal leakage of dialysis fluid, another sign of this complication is the presence of pain in the iliac area, equated with Mittelschmerz present in some patients, although in most patients, the course of this complication is usually asymptomatic [6].

Obstruction of the catheter by oviductal fimbriae is a potentially serious complication because manipulation without direct vision can cause tissue detachment and significant hemorrhage, with consequent fallopian tube damage and subsequent infertility [6].

Fimbriectomy can be considered in the surgical management of patients with this complication to avoid recurrence, being a reasonable alternative in elderly patients; however, it should be discussed and debated in the case of girls. In the case of repeated obstruction, it is usually necessary to perform a salpingectomy [8].

When faced with a patient with obstructed flow, maneuvers have been established to try to recanalize the patient, such as active mobilization of the patient, induction of peristalsis with enemas, manipulation of the catheter with a rigid wire, and infusion of fibrinolytic agents such as urokinase; however, all have shown limited success [4,7].

In a patient who does not improve with conservative measures, early surgery and careful surgical exploration are required. In most cases, it is usually necessary to remove or replace the catheter [4,6].

In the case of our patient, she presented with decreased outflow of the peritoneal catheter and severe abdominal pain in the right iliac fossa. According to the literature, most patients are asymptomatic. An abdominal CT scan was performed, which ruled out a kinked or bent catheter and showed an orientation towards the right iliac fossa. Due to the presence of intense pain and signs of peritoneal irritation, it was decided to perform open surgery due to the suspicion of visceral entrapment by the catheter, since, according to current evidence, conservative measures do not improve this complication, having only limited success in cases of obstruction by fibrin thrombi. During surgery, the fallopian tube was removed without damage or hemorrhage, and it was found that the catheter was in optimal condition, so it was relocated and there was no need to replace it.

## Conclusions

One of the most common complications of peritoneal dialysis is catheter obstruction, which can occur due to intra-abdominal organs, the most frequent being the omental sheath. Obstruction by the fallopian tube and its fimbriae is a very infrequent presentation, which, together with catheter dysfunction, can be asymptomatic, cause abdominal pain, or result in vaginal leakage of dialysis fluid. When this complication is suspected, the catheter should not be manipulated blindly, since it can cause significant bleeding, damage to the oviduct, and infertility. The release of the fallopian tube and repositioning of the catheter can be performed by open or laparoscopic surgery, and fimbriectomy to avoid recurrence can be considered in elderly patients or those with satisfied parity.
